# Ipilimumab administration for advanced melanoma in patients with pre-existing Hepatitis B or C infection: a multicenter, retrospective case series

**DOI:** 10.1186/s40425-014-0033-1

**Published:** 2014-10-14

**Authors:** Sowmya Ravi, Kristen Spencer, Mary Ruisi, Nageatte Ibrahim, Jason J Luke, John A Thompson, Keisuke Shirai, David Lawson, Heddy Bartell, Ragini Kudchadkar, Ngoc Thi Gunter, Janice M Mehnert, Evan J Lipson

**Affiliations:** The Johns Hopkins University School of Medicine and Sidney Kimmel Comprehensive Cancer Center, 1550 Orleans St., Rm. 507, Baltimore, MD 21287 USA; Rutgers Cancer Institute of New Jersey, 195 Little Albany Street, New Brunswick, NJ 08901 USA; Bristol-Myers Squibb, Oncology Global Clinical Research, Route 206 and Provinceline Road, Princeton, NJ 08543 USA; Dana-Farber Cancer Institute Melanoma Disease Center, Harvard Medical School, 450 Brookline Ave, Boston, MA 02215 USA; Dana-Farber Cancer Institute Melanoma Disease Center, Center for ImmunoOncology, Harvard Medical School, 450 Brookline Ave, Boston, MA 02215 USA; Seattle Cancer Care Alliance, University of Washington, 825 Eastlake Ave. East, Seattle, WA 98109 USA; Division of Hematology/Oncology, Medical University of South Carolina, 173 Ashley Ave., Basic Science Building, Suite 102, Charleston, SC 29425 USA; The Winship Cancer Institute of Emory University School of Medicine, 1365C Clifton Road, Atlanta, Georgia 30322 USA; Bristol-Myers Squibb. Oncology Medical Strategy, 777 Scudders Mill Road, Plainsboro, NJ 08536 USA

**Keywords:** Ipilimumab, Melanoma, Hepatitis B, Hepatitis C

## Abstract

Ipilimumab is a fully human, monoclonal antibody directed against Cytotoxic T Lymphocyte Antigen-4 (CTLA-4) that has demonstrated a survival benefit and durable disease control in patients with advanced melanoma. Ipilimumab is associated with potentially serious immune-related adverse events, including autoimmune hepatitis. Because clinical trials of ipilimumab excluded patients with pre-existing hepatitis B or C infection, there is a paucity of data on the safety of ipilimumab administration to that patient population. Here, we report the largest case series to date of patients with hepatitis B or C who received ipilimumab for advanced melanoma. Two of the nine patients described in this case series experienced fluctuations in their liver function tests (LFTs) and were subsequently treated with corticosteroids. Although this is a small series, the rate of hepatotoxicity appears similar to what has been seen in the general population treated with ipilimumab, and the ability to administer ipilimumab did not appear to be affected by concomitant hepatitis B or C infection. The use of ipilimumab in patients with metastatic melanoma who have pre-existing hepatitis can be considered among other therapeutic options.

## Background

The US Food and Drug Administration’s approval of ipilimumab (Yervoy, Bristol-Myers Squibb, Princeton, NJ) in 2011 heralded a new era in the treatment of advanced melanoma. A fully human monoclonal antibody against Cytotoxic T Lymphocyte Antigen-4 (CTLA-4), ipilimumab demonstrated a survival benefit and durable disease control in randomized, controlled phase III clinical trials [1-3]. However, those trials and others performed during the pre-marketing drug development of ipilimumab excluded patients with pre-existing hepatitis B or C. Moreover, ipilimumab administration is associated with serious (grade 3/4) adverse events with potential immune-related causality, including autoimmune hepatitis. Although the risk of reactivation of hepatitis B (HBV) or C (HCV) in infected patients is well-described in patients receiving cytotoxic chemotherapy [4,5], there is a paucity of data on the safety of administering ipilimumab or other immunotherapies to that patient population [6,7]. Here, we report the largest case series to date of patients with hepatitis B or C who received ipilimumab for advanced melanoma. All patients received ipilimumab at the FDA-approved dose of 3 milligrams per kilogram of body weight. Cases are described in detail below and are summarized in Table 1.

## Case presentations

**Table 1:** **Clinical summary of patients with pre-existing Hepatitis B and C treated with ipilimumab**

**Case No.**	**Age (yrs)**	**Sex**	**Liver metastases?**	**Type of Hepatitis**	**Evidence of liver fibrosis?**	**Therapy for metastatic melanoma prior to ipilimumab**	**LFTs prior to administration of ipilimumab**	**Change in LFTs during Ipilimumab**	**Best Response to Ipi**	**Comments**
1	65	M	N	HCV (active)	Y	High dose Interleukin-2	Gr 1 AST, Gr 1 ALT	None	PD	HCV viral load increased four-fold after IL-2, ipilimumab and temozolomide
2	56	M	N	HCV (active)	Unknown	Stereotactic Radiotherapy, then WBRT	AST & ALT WNL	None	PD	Possible drug-related hepatitis detected 2 months after receiving 4 doses of ipilimumab
3	43	M	N	HCV (active)	N	Temozolomide	Gr 2 AST, Gr 2 ALT	Normalized by cycle 4	Mixed response	HCV viral load decreased to undetectable levels after 4 doses of ipilimumab
4	71	M	N	HCV (active)	Y	None	Gr 1 AST, Gr 1 ALT	Gr 2 AST, Gr 3 ALT after cycle 3	SD	HCV viral load decreased 5-fold to 408,000 IU/mL after 3 doses of ipilimumab; ocular melanoma
5	56	M	(multiple tumors involving 25-75% of liver, largest = 6.4 cm)	HBV (inactive)	Unknown	None	Gr 1 AST, ALT WNL	None	PD	Concurrent administration of entecovir for prophylaxis against HBV reactivation
6	60	M	N	HBV (active)	Unknown	high-dose interleukin-2, talimogene laherparepvec and dacarbazine	AST & ALT WNL	None	PD	Tenofovir administration prior to ipi brought HBV viral load from 2950 to 41 IU/mL; became undetectable and remained so throughout ipilimumab
7	42	F	(multiple tumors involving 30-40% of liver, largest = 2.7 cm)	HBV (inactive)	Unknown	None	AST & ALT WNL	None	PD	HBV viral load undetectable prior to starting ipilimumab
8	56	M	N	HBV (active)	Unknown	None	AST & ALT WNL	None	PD	Entecavir administration prior to ipi brought HBV viral load from 9560 to 454 IU/mL; became undetectable and remained so throughout ipilimumab
9	71	M	N	HBV (active)	Cirrhosis noted on CT; no confirming path findings	None	Gr 1 AST, ALT WNL	None	SD	HBV viral load 700 IU/mL prior to starting ipilimumab

Table 1 (Gr, Grade of toxicity as measured by Common Terminology Criteria for Adverse Events (CTCAE) version 4.0; AST, aspartate aminotransferase; *ALT*, Alanine aminotransferase; *LFT*, Liver function tests; *PD*, Progressive disease; *Ipi*, ipilimumab; *SD*, Stable disease)).

Case 1: A 65-year-old man presented with metastatic melanoma involving his lungs and mediastinal and neck lymph nodes; he had no detectable hepatic metastases. The patient had contracted HCV (genotype 1a) approximately twenty five years prior to his melanoma diagnosis, though his hepatitis was never treated. A liver biopsy performed in 2000 demonstrated periportal fibrosis without bridging as well as mild inflammatory activity. Pathologic findings from a repeat liver biopsy in 2005 were unchanged. His melanoma was initially treated with high-dose interleukin-2 (IL-2) in May 2011. After two cycles, his melanoma had regressed, but he developed obstructive jaundice secondary to cholelithiasis with gallbladder sludge, requiring a laparoscopic cholecystectomy. Serologic evaluation around the time of surgery (August 2011) revealed the following: HCV viral load: 690,000 IU/mL, hepatitis A IgM negative, HBV core (HBc) IgG and IgM negative, hepatitis B surface antigen (HBsAg) negative. A liver biopsy performed during surgery revealed cirrhosis with mild, non-specific inflammation and focal hemosiderin accumulation. He received two more cycles of IL-2, but his melanoma subsequently progressed and he began treatment with ipilimumab in November 2011. During and after ipilimumab administration, hepatic transaminases remained unchanged from baseline, with grade 1 elevations. Although the patient tolerated 4 cycles of ipilimumab without drug-limiting toxicities, he experienced disease progression and was transitioned to temozolomide. After three cycles of temozolomide, HCV viral load had increased four-fold to 2,720,000 IU/mL. The patient expired sixteen months after the initiation of ipilimumab from progression of his melanoma.

Case 2: A 56-year-old man with HCV was found to have stage IIIA cutaneous melanoma. A local recurrence in June 2010 was treated with excision and axillary lymph node dissection. Laboratory values at that time revealed an aspartate aminotransferase (AST) of 45 IU/L (normal range 0-40 IU/L), a normal alanine aminotransferase (ALT) (0-55 IU/L), a negative HBsAg, a negative HBc IgM, a positive HCV antibody and an HCV viral load of 103,160 IU/mL. He did not receive systemic treatment for his hepatitis. Subsequent restaging scans demonstrated new metastases in lymph nodes, muscle and brain. He underwent stereotactic radiosurgery for his brain lesion followed by two cycles of temozolomide. During systemic therapy with temozolomide, the patient’s hepatic transaminases rose: AST 254 IU/L, ALT 325 IU/L. Shortly thereafter, new brain and soft tissue lesions appeared. Whole-brain radiotherapy was administered, followed by initiation of ipilimumab in April 2011. Immediately prior to ipilimumab administration, hepatic transaminases had normalized (AST 29 IU/L, ALT 17 IU/L), and HCV viral load was 3,070 IU/mL. He underwent four cycles of ipilimumab without significant changes in liver function tests. The patient experienced progressive disease five months after initiating ipilimumab, at which time HCV viral load was 2,279,050 IU/mL, and AST and ALT had increased to 208 and 119 IU/L, respectively. Because of the potential for immune-related causality, corticosteroid therapy was administered, with improvement of hepatic transaminases (AST 117, ALT 36 IU/L). The patient then enrolled in a phase I clinical trial of a pan-RAF kinase inhibitor due to progression of disease, and was maintained on steroids during the course of experimental therapy. His course was marked by liver function test fluctuations (AST 35-117, ALT 28-132 IU/L) as well as adrenal insufficiency after decreasing the patient’s dose of corticosteroids. Ten months after initiating ipilimumab, HCV viral load was >69,000,000 IU/mL, however, his hepatic transaminase levels subsequently normalized. Although his viral load and LFTs were discordant, the patient was approaching hospice care at this time and further investigation of the significance of these findings was postponed. Two months later, the patient died from progressive melanoma. Of note, this case was previously reported in detail but summarized here to augment the current case series [7].

Case 3: A 43-year-old man presented with metastatic melanoma involving axillary, hilar and pericardiac lymph nodes and soft tissue adjacent to the umbilicus, but no hepatic lesions. He was treated with six cycles of temozolomide, during which liver transaminases became elevated: AST 85 IU/L (normal 8-48 IU/L), ALT 164 IU/L (10-40 IU/L). The transaminitis persisted after discontinuation of temozolomide. An abdominal computed tomography (CT) scan showed no evidence of intra-abdominal metastases or hepatic cirrhosis; however serologies revealed HCV with a viral load of 398,938 IU/mL, genotype 1a. The patient tolerated four cycles of ipilimumab from April 2012 to July 2012 without interruption or complication. He had a mixed response to ipilimumab, but never progressed. Hepatic transaminases normalized by cycle 4, and HCV viral load was detectable at <12 IU/mL approximately four weeks later. Post-therapy the patient’s viral load began to climb: 1,378 IU/mL at eight weeks and 1,558 IU/mL at fourteen weeks after the fourth cycle of ipilimumab was administered. A remaining melanoma lesion in the right hilum was irradiated, and subsequent imaging with positron emission tomography (PET)/CT in January 2014 and CT in July 2014 demonstrated no evidence of disease. Since completing ipilimumab, HCV viral load has remained stable with normal LFTs. Of note, this case was previously reported in detail but summarized here to expand the current case series [6].

Case 4: A 71-year-old man presented with metastatic ocular (choroidal) melanoma without hepatic involvement. His primary lesion had been treated thirteen years earlier with right orbital enucleation. Three years after enucleation he was diagnosed with HCV but did not require treatment. Evaluations for hepatitis A, HBV and HIV were negative. Serologic evaluation prior to initiation of ipilimumab revealed HCV viral load of 2,310,000 IU/mL and grade 1 AST and ALT. Three weeks after one cycle of ipilimumab, the patient was found to have grade 2 and 3 elevations of AST and ALT, respectively. Ipilimumab was held and high-dose prednisone was administered. HCV viral load had decreased to 408,000 IU/mL. Liver biopsy demonstrated chronic, active Scheuer grade 2 hepatitis and Scheuer stage 2 periportal fibrosis. There was no evidence of associated autoimmunity; a panel of serologic markers associated with autoimmune hepatitis (anti-nuclear cytoplasmic antibody, IgG; double stranded DNA antibody, IgG; F-actin antibody, IgG; anti-soluble liver antigen, IgG; mitochondrial M2 antibody, IgG; liver kidney microsome antibody; and anti-neuronal nuclear antibody) was negative. Extractable nuclear antigen screen was equivocal and was not pursued. His serum IgG was mildly elevated at 1630 mg/dL (normal range 694-1,618). As there was no evidence of autoimmune hepatitis, his prednisone was discontinued. Magnetic resonance imagining (MRI) of the abdomen revealed no evidence of malignancy. The patient’s ALT and AST returned to grade 1, and, after a brief dose delay, ipilimumab was restarted with subsequent normalization of hepatic transaminases. Five months after initiation of ipilimumab the patient’s melanoma was stable and HCV viral load was 108,000 IU/mL. Eight months after initiation of ipilimumab, however, a bronchoscopy and PET/CT scan both showed progression of disease without hepatic involvement. Shortly thereafter, serum AST and ALT became elevated (grade 1). HCV viral load measured 11 months after initiation of ipilimumab increased to 653,000 IU/mL. The patient was treated with temozolomide, which was discontinued due to thrombocytopenia. He subsequently relocated and was lost to follow-up.

Case 5: A 56-year-old man with a history of HBV presented with stage IV melanoma involving the liver (multiple tumors, largest = 6.4 cm), pancreas, kidney, bones and mediastinal lymph nodes. At the time of ipilimumab administration, the patient’s serum AST was 61 IU/L (normal range = 8-48) and ALT was normal at 25 IU/L. His bilirubin was mildly elevated at 1.57 mg/dL (0.1-1.2). Serologies revealed the following: HBsAg negative; HBV DNA undetectable by polymerase chain reaction (PCR); HBc reactive; HBV surface antibody negative, suggesting an inactive carrier state. The patient did not have a liver biopsy, so it is unclear if he had fibrosis at the time of ipilimumab administration. The patient completed a total of 3 cycles of ipilimumab, along with concurrent administration of entecavir for prophylaxis against HBV reactivation. Though his liver function tests remained normal throughout therapy, the final cycle of ipilimumab was withheld due to clinical decompensation consisting of progressive fatigue and pain due to bone and liver metastases. The patient died of progressive melanoma shortly after receiving his third cycle of ipilimumab therapy, at which time hepatic transaminases were within normal limits.

Case 6: A 60-year-old man presented with stage IV melanoma including multiple subcutaneous metastases. He had previously undergone adjuvant therapy with interferon alfa (IFNα) after surgical resection of his primary lesion, as well as radiation therapy after resection of a local recurrence. Prior therapies for metastatic disease included high-dose IL-2, talimogene laherparepvec and dacarbazine with disease progression after each. Two months prior to the initiation of ipilimumab, the patient’s serum AST and ALT were within normal limits with a reactive Hepatitis B e-Antibody (anti-HBe), nonreactive Hepatitis B e-Antigen (HBeAg), and HBV viral load of 43,100 IU/mL. Without intervening therapy, HBV viral load fell to 2,950 IU/mL ten days later. Tenofovir therapy was initiated. After four days of treatment the patient’s viral load was 41 IU/mL. Four cycles of ipilimumab were administered, which were well-tolerated and produced no LFT abnormalities. One month later, HBV viral load was undetectable. The patient expired four months after initiation of ipilimumab from progressive melanoma. Of note, this case was previously reported in detail but summarized here to expand the current case series [7].

Case 7: A 42-year-old Vietnamese woman, who had undergone resection of a mucosal (anal) melanoma two years earlier, presented with metastatic melanoma involving liver and bone. Serologies prior to treatment with ipilimumab revealed an undetectable HBV DNA by PCR, a negative HBeAg and positive anti-HBe, indicating viral exposure and clearance. Liver function studies were within normal limits prior to initiation of ipilimumab and remained normal throughout all 4 cycles of therapy. Though the patient did not experience any ipilimumab-related toxicity, radiologic scans performed one month after the final cycle demonstrated progression of disease.

Case 8: A 56-year-old Venezuelan man with HBV, who had previously received adjuvant IFNα after resection of a primary melanoma (cKIT mutant), presented with metastatic disease including pancreatic and splenic metastases. Prior to the initiation of ipilimumab, the patient’s HBV DNA level was 9560 IU/mL with normal liver function tests. The patient stated that he had a liver biopsy in South America which was within normal limits; however this was not confirmed by repeat liver biopsy, although his radiographic imaging from the US does not show evidence of fibrosis. The patient was administered entecavir 0.5 mg daily, after which the viral load fell to 454 IU/mL. Ipilimumab was initiated 2 weeks later and entecavir was continued. The patient’s viral load became undetectable, and his LFTs remained within normal limits throughout the course of treatment. Although he completed 4 cycles of ipilimumab without significant complications, post-therapy radiologic scans demonstrated progressive disease. He was subsequently treated with temozolomide, then nilotinib, again without hepatic complications.

Case 9: A 71-year-old man presented with stage IV melanoma. He had received adjuvant IFNα after his initial melanoma diagnosis and surgical resection seven years prior, which coincided with a diagnosis of HBV. Hepatic transaminases prior to administration of IFNα were normal, though IFNα was held several times due to grade 2 and 3 elevations of ALT and AST. Three years prior to administration of ipilimumab, the patient was initiated on HBV treatment with tenofovir after serologic markers revealed a HBV viral load of >200,000,000 IU/mL and CT imaging demonstrated cirrhotic morphology and small volume ascites. One year into tenofovir therapy, HBV viral load had decreased to 1900 IU/mL. Two years later, laboratory evaluation immediately prior to initiation of ipilimumab revealed normal ALT, grade 1 elevation of AST and HBV viral load of 700 IU/mL. Hepatic transaminases remained stable throughout ipilimumab induction therapy, which resulted in stable disease for seven months. The patient’s melanoma then progressed, which was again treated with ipilimumab. ALT remained normal, AST remained grade 1; however the patient’s melanoma progressed five months later with no evidence of hepatic involvement. The patient remains on tenofovir at the time of this publication.

## Conclusions

Although ipilimumab has undergone extensive clinical testing in patients with melanoma [8], its use in patients with infectious co-morbidities remains poorly characterized. Patients bearing similarity to the population broadly studied during clinical development often receive all four cycles of ipilimumab without evidence of autoimmune toxicity. However, other patients experience adverse events with potential immune-related causality, [otherwise “immune-related adverse events”] that can be dose limiting and cause significant morbidity [9]. Indeed, the two large phase III trials conducted prior to the drug’s approval by the FDA excluded patients receiving immunosuppressive medications, those with conditions requiring the long-term use of systemic corticosteroids and those with acute or chronic viral hepatitis. Since the side effect profile observed with ipilimumab differs significantly from that of standard cytotoxic chemotherapy due to its unique mechanism of action, understanding its relative safety in patients with hepatitis infection is crucial in helping to establish treatment guidelines. This is especially important in light of the fact that immune-mediated hepatotoxicity, a known adverse effect of ipilimumab, is of particular concern in patients with concomitant viral hepatitis. This concern arises from the theoretical risk of hepatitis B/C reactivation and the potential for immune-mediated liver damage in patients whose hepatic function may be compromised at baseline.

**Figure 1:**
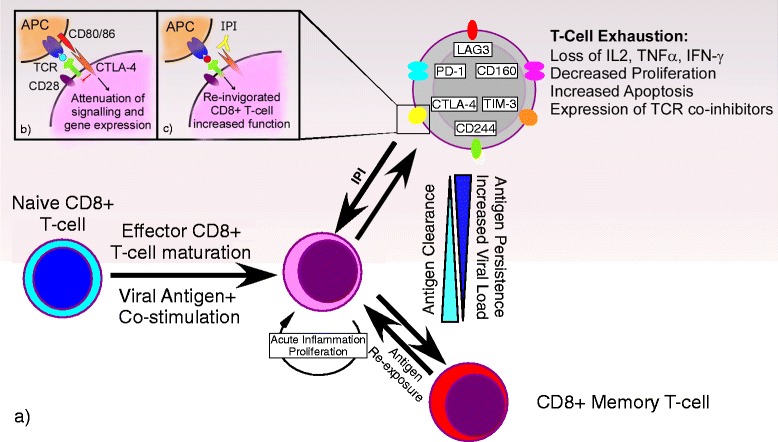
**The process of T cell exhaustion in chronic viral infection and possible effects of ipilimumab. a)** After the introduction of initial antigen (infection), naive CD8+ T cells are primed by antigen and, with concomitant stimulation and ongoing inflammation, mature into effector CD8+ T cells. As the antigen (infection) clears, a subset of the effector CD8+ T cells further differentiate into memory CD8+ T cells with the ability to produce cytokines such as IL-2, tumor necrosis factor (TNF) and interferon-γ (IFN-γ), then degranulate, proliferate and self-rejuvenate. In the event of chronic antigen exposure (infection) and subsequent increasing viral load and persistence of antigen (infection), mature effector CD8+ T cells proceed through a hierarchical process of exhaustion, loss of effector functions including the ability to produce cytokines and degranulate, the progressive expression of inhibitory receptors, and the loss of the ability to proliferate and self-rejuvenate and elimination. These processes culminate in the loss of virus-specific CD8+ T cell responses. **b)** Cytotoxic T-Lymphocyte Antigen 4 (CTLA-4) is an inhibitory receptor expressed on the surface of exhausted CD8+ T cells. It is analogous to, but serves the opposite function of, the CD28 T cell receptor also found on the surface of these cells. Activated antigen presenting cells (APC) loaded with antigen express CD80/86, which can either bind CD28 resulting in a stimulatory signal, or bind to CTLA-4 resulting in attenuation of further intracellular signaling and gene expression and ultimately CD8+ T cell response. **c)** Ipilimumab is a monoclonal antibody that targets the CTLA-4 receptor on CD8+ T cells. It functions by binding the CTLA-4 receptor, thus preventing CD80/86-CTLA-4 binding and the resultant CD8+ T cell attenuation, thereby halting and potentially reversing the process of CD8+ T cell exhaustion.

Chemotherapy-induced reactivation of hepatitis B or C that results in significant hepatic decompensation is well described [4,5]. Higher serum viral load prior to the initiation of therapy may influence risk of reactivation, and high pretreatment HBV DNA levels have been shown to correlate with poor tolerance to cytotoxic chemotherapy [10,11]. Several of these issues are being investigated in ongoing clinical trials. (NCT01658878)A theoretical risk of viral reactivation exists with the use of ipilimumab because of the presence of CTLA-4 on T regulatory (Treg) cells. This sub-population of immunoregulatory T cells suppresses the activation and effector function of CD4+ and CD8+ T cells [12–14]; thus, activating Tregs by blocking CTLA-4 may further impair the ability of T cells to keep viral hepatitis suppressed [15]. On the other hand, HCV viral replication is associated with “exhausted” CD8+ T cells, which express immune-inhibitory receptors, such as CTLA-4 and Programmed Death-1 (PD-1) (see Figure 1; [16]). Blockade of one or more of these immune checkpoints has demonstrated a decrease in HCV viral load and a reversal of HCV-specific T cell exhaustion which may stimulate an antiviral response [6,17–19]. Indeed, this phenomenon may underlie the decrease in viral load observed after administration of ipilimumab in case #3, described above. A similar mechanism may have led to the activation and proliferation of tumor-specific T cells, culminating in a durable anti-tumor response.

Autoimmune hepatotoxicity is a side effect of ipilimumab and of immune checkpoint inhibitors as a class. In a phase III trial combining ipilimumab with dacarbazine as first-line therapy for advanced melanoma, 78 of 247 (31.6%) patients who received both drugs experienced grade three or four immune-mediated hepatitis [3]. Of note, because dacarbazine monotherapy can cause hepatotoxicity, interpretation of these results was challenging. In a study of twenty one patients with hepatocellular carcinoma and chronic hepatitis C who received tremelimumab, another CTLA-4 blocking antibody, 45% experienced a transient grade three or higher rise in liver transaminases after the first dose [17]. Similarly, evidence of drug-related hepatic toxicity has also been reported with PD-1 antibodies [20,21]. In lieu of these risks, recommendations for administration of ipilimumab include monitoring of hepatic function tests prior to each dose and the rapid initiation of corticosteroid therapy in patients with ≥ grade 3 elevations of hepatic transaminases that are unrelated to melanoma progression [9]. In such patients, some investigators assess levels of serum autoimmune markers such as anti-nuclear antibodies, anti-smooth muscle antibodies and anti-nuclear cytoplasmic antibodies. Two of the nine patients, described in this report, both of whom had chronic HCV infection, experienced fluctuations in LFTS and were treated with corticosteroids. Although this is a small series, the rate of hepatotoxicity appears similar to what was seen in the general population treated with ipilimumab, and the ability to administer ipilimumab did not appear to be affected by concomitant hepatitis B or C infection.

There remain limited published data describing the safety of ipilimumab administration to patients with metastatic melanoma who have pre-existing HBV or HCV. Although the nine patients described herein (five with HBV, four with HCV) represent the largest case series on this topic, our report is limited by the retrospective collection of data from all patients. For instance, viral load information was not available on every patient treated to allow for uniform comparisons. The use of ipilimumab in patients with metastatic melanoma who have pre-existing hepatitis can be considered among other therapeutic options. Careful attention to baseline hepatic function and additional comorbidities is critical, as is close monitoring of LFT trends while patients are receiving therapy and consultation with specialists in chronic liver disease. As in patients treated with chemotherapy, patients with Hepatitis B infection may be candidates for concomitant HBV suppression with antiviral therapies, an additional risk mitigation strategy. Although a concomitant diagnosis of HBV or HCV is not an absolute contraindication to the administration of ipilimumab, a more thorough characterization of the drug’s effect on hepatitis, in particular viral load suppression, requires further evaluation in prospective studies.

## Consent

Written informed consent was obtained from the patients for publication of this Case series. A copy of the written consent is available for review by the Editor-in-Chief of this journal.

## Abbreviations

ALT: Alanine aminotransferase
AST: Aspartate aminotransferase
CT: Computed tomography
CTLA-4: Cytotoxic T Lymphocyte Antigen-4
HBc: Hepatitis B core Antibody
anti-HBe: Hepatitis B e-Antibody
HBeAg: Hepatitis B e-Antigen
HBsAg: Hepatitis B surface antigen
HBV: Hepatitis B Virus
HCV: Hepatitis C Virus
IFNα: Interferon alfa
IL-2: Interleukin-2
LFTs: Liver function tests
MRI: Magnetic resonance imagining
PR: Partial response
PCR: Polymerase chain reaction
PET: Positron emission tomography
PD-1: Programmed Death-1
PD: Progressive disease
SD: Stable disease
Treg cells: T regulatory
